# Short-term severe drought influences root volatile biosynthesis in eastern white pine *(Pinus strobus L)*

**DOI:** 10.3389/fpls.2022.1030140

**Published:** 2022-10-26

**Authors:** Umashankar Chandrasekaran, Siyeon Byeon, Kunhyo Kim, Seo Hyun Kim, Chan Oh Park, Ah reum Han, Young-Sang Lee, Hyun Seok Kim

**Affiliations:** ^1^ Department of Agriculture, Forestry and Bioresources, College of Agriculture and Life Sciences, Seoul National University, Seoul, South Korea; ^2^ Research Institute of Agriculture and Life Sciences, College of Agriculture and Life Sciences, Seoul National University, Seoul, South Korea; ^3^ Division of Basic Research, National Institute of Ecology, Seocheon-gun, South Korea; ^4^ Interdisciplinary Program in Agricultural and Forest Meteorology, College of Agriculture and Life Sciences, Seoul National University, Seoul, South Korea; ^5^ National Center for Agro Meteorology, Seoul, South Korea

**Keywords:** drought stress, starch, terpenoids α-pinene, limonene, limonene oxide, root

## Abstract

Climate change-related drought stress is expected to shift carbon partitioning toward volatile organic compound (VOC) biosynthesis. The effect of drought stress on VOC synthesis remains unknown in several tree species. Therefore, we exposed eastern white pine (*Pinus strobus*) plants to severe drought for 32 days and performed physiological analysis (chlorophyll content, leaf water content, and root/shoot index), biochemical analysis (non-structural carbohydrates, proline, lipid peroxidation, and antioxidant assay), and total root VOC analysis. Drought stress decreased the relative water and soil moisture contents. Root proline accumulation and antioxidant activity increased significantly, whereas leaf chlorophyll synthesis and fresh weight decreased significantly in drought-treated plants. A non-significant increase in sugar accumulation (leaves and roots), proline accumulation (leaves), antioxidant activity (leaves), and lipid peroxidation (leaves and roots) was observed in drought-treated plants. Drought stress caused a non-significant decline in root/shoot ratio and starch accumulation (leaves and roots) and caused a significant increase in root abscisic acid content. Drought-treated plants showed an increase in overall monoterpene synthesis (16%) and decline in total sesquiterpene synthesis (3%). Our findings provide an overall assessment of the different responses of VOC synthesis to severe water deficit that may help unravel the molecular mechanisms underlying drought tolerance in *P. strobus*.

## Introduction

Global warming-mediated climate change is expected to increase the frequency of water shortage, heat stress, and increased soil salinity. Drought stress significantly decreases the growth and yield of most plants (Polle et al., 2019; dos Santos et al., 2022). It has been speculated that the expected environmental changes will strongly affect growth, reproduction, defense, and communication processes of plants (Zhang et al., 2020; Ullah et al., 2021). Drought is a multifaceted environmental restraint that can elicit tree responses from the molecular to the forest level (Li et al., 2021; Bradford et al., 2022). It affects soil nutrient availability for plants and the uptake capacities of plant roots, and consequently influences the nutrient status of trees (Bista et al., 2020; Tan et al., 2021). Drought stress causes elevated production of reactive oxygen species (ROS) in plants (Vijayaraghavareddy et al., 2022). To survive drought stress, trees respond at morphological (decreased leaf area and altered leaf structure), physiological (stomatal closure, activation of autotrophic respiration, and accumulation of osmolytes and stress-resistant proteins), and molecular (altered gene expression patterns) levels (Moran et al., 2017; Semerci et al., 2017; Haas et al., 2021).

Plants have developed various mechanisms to perceive, transduce, and respond to ROS signals to protect themselves from soil pathogens and from abiotic stress-derived damage, such as drought, salinity, and extreme temperatures. Tree root systems are key components of forest ecosystems which influence water and nutrient uptake and function as sensors for water-deficit conditions and send signals to shoots above ground (Quan and Ding, 2017; Yang et al., 2021). Roots play a crucial role in protecting plants against oxidative stress by mainly inducing the biosynthesis of volatile terpenoids, which is thought to quench ROS (Possell and Loreto, 2013; Kleiber et al., 2017). Further, plant roots can improve pest control *via* these chemical signals (Delory et al., 2016). Volatiles emitted by roots act as anti-microbial or anti-herbivore substances in contributing to belowground defence (Schenkel et al., 2015).

Tree roots synthesize volatile organic compounds (VOCs) *via* secondary metabolism, which mainly includes production of terpenoids and fatty acid derivatives, and they serve as an important regulator of plant resistance to attenuate stress (Kleiber et al., 2017; Gfeller et al., 2019). Terpenoids, mainly monoterpenes and sesquiterpenes, are thought to play important roles in abiotic (heat and oxidative stress) and biotic stress defense mechanisms (Kleiber et al., 2017; Bertamini et al., 2019). In addition, root terpenoids play a crucial role in the defense against herbivores and pathogens (Sharma et al., 2017; Lackus et al., 2018). Previous studies have analyzed the VOCs of *Pinus* species including *Pinus tabuliformis, Pinus bungeana, Cedrus deodara, Pinus thunbergii (* Ji and Ji, 2021 *)*, *Pinus densiflora* (Otaka et al., 2017), *Pinus halepensis* (Blanch et al., 2007), *Pinus mugo* (Celiński et al., 2015), *Pinus sylvestris* (Szmigielski et al., 2011), *Pinus massoniana* (Quan and Ding, 2017), with a majority of the studies focusing on needles rather than roots.

While few studies have focused on root terpenoids in tree species (Kleiber et al., 2017; Otaka et al., 2017; Quan and Ding, 2017; Chang et al., 2021), there is a complete lack of knowledge on the relationship between root terpenoids and water deficit conditions. More importantly, it is currently unknown whether terpenoid biosynthesis mitigates or alleviates drought stress in tree species *Pinus strobus*. Eastern west pine (P. strobus L), native to eastern North America, is an ecologically and economically important species of temperate white pine ecosystems. P. strobus was imported to Korea in the early 1970s and represents an essential tree species to preserve the Korean ecosystem. Pine trees are very important as they represent one of the dominant species of global forests as they provide food and cover for small animals and birds. Studies on several pine species have revealed the vulnerability of these species to abiotic stress, especially drought stress (Mitchell et al., 2013; Quan and Ding, 2017; Asbjornsen et al., 2021; Lee et al., 2021b). However, Korean pine species show seasonal variations in their drought response (Song et al., 2020; Lee et al., 2021a). A previous study on the response of P. strobus to drought only evaluated the woody growth response and threshold dynamics (Asbjornsen et al., 2021). Therefore, there is a need to conduct a drought response study in young P. strobus saplings to understand the early physiological and biochemical responses.

Soil water availability represents a major environmental constraint in Korean forests, and estimates suggest that the decline in total rainfall will be drastic in the near future (Kim et al., 2014; Nam et al., 2015) Under such conditions, it is likely that young Pinus plants will experience increasing water deficit stress in Korean natural communities. There is a considerable need to elucidate the response of *P. strobus* seedlings to drought to develop strategies for the preservation of tree growth and survival owing to this particular environmental threat at early stages. Therefore, we evaluated the short-term severe drought response in *P. strobus* saplings in relation to their root VOC biosynthesis in the present study.

The main aims of this study were to investigate the physiological characteristics (plant height, chlorophyll content, relative water content, fresh weight, root length, and root color diameter), biochemical changes (proline biosynthesis, non-structural carbohydrate (NSC) content, lipid peroxidation, radical scavenging activity, and abscisic acid (ABA) biosynthesis), and root volatile biosynthesis patterns in *P. strobus* after a severe drought stress period.

## Materials and methods

### Experimental design and treatment

The study was conducted at an experimental site on mountain Jiri (E 127°27′09″ N 35°16′50″, elevation 1,289 m above sea level) in Gurye, South Jeolla Province, Republic of Korea. The mean annual temperature in the area is 13.4°C, the maximum summer temperature is approximately 38°C, and the average annual precipitation is 1,345.7 mm, based on data collected between 1997–2018 (Korea Meteorological Administration, 2018). A frame of galvanized metal allowing sufficient air circulation was constructed over the study plots, and transparent Plexiglas roof allowing 91% light transmission was installed at a height of 3 m. Two pot (circular, 16 cm height × 16 cm top diameter × 12 cm bottom diameter) treatments were applied: control (100% natural precipitation) and drought (20% precipitation) (**Supplementary Figure S1**). The drought treatments were applied by excluding natural precipitation by opening only 20% (severe) of the Plexiglas roof area (Bhusal et al., 2021). The pots were placed exactly under the wider opening region for control and narrow region for inducing drought stress (**Supplementary Figure S1**). The seedlings for each group (5 + 5) were kept at two different spots under the same plexiglass roof. The soil consisted of a mixture of sandstone, sand, mudstone, and gravel, with a pH of 6.5. The treatment was performed up to 32 days using three-year old P. strobus saplings (10 replicates). Precipitation (mm) was measured using a HOBO S-RGF sensor (Onset Computer Corporation, Bourne, MA, USA) throughout the study period. The study was conducted at the end of the growing season (1 November 2021 to 2 December 2021).

### Morphological traits

The height, root length (primary root), and root collar diameter (one spot with two different angles) of all plants were measured using a millimeter tape and Vernier caliper (Mitutoyo, Kawasaki, Japan), respectively. Leaf FW (five leaves per plant) was measured using a high precision balance (Sartorius-BCE64i, Göttingen, Germany). The measurements were recorded twice during the experiment: at the beginning and at the end of the drought stress period. The root/shoot (R/S) ratio was estimated as follows:


$$R/S\;ratio\;(g\;DW^{-1})=DW\;of\;the\;roots/DW\;of\;the\;shoots$$

### Soil moisture content

Soil from all the pots was collected using a stainless-steel metal cup (3 × 3 cm, SZ metals, Korea). Soil samples were collected at five-centimeter-depth after removing the litter layer, weighed, and dried in an oven at 80°C for 72 h before measuring the DW. Moisture content of the soil sample was expressed in percentage according to the formula:


Soil moisture content (SMC)(%) = [(FW – DW)/DW] × 100

### Leaf relative water content

Relative water content (RWC) was measured using 10 fully expanded needles from the current year’s lateral branch. To evaluate leaf RWC, the FW, DW, and turgid weights (TWs) of the leaves were measured after 32 days after treatment (DAT). Ten replicates for each treatment were used for measuring RWC and the percentage of RWC was calculated using the formula:


RWC(%)=(FW–DW)/(TW–DW)×100

### Chlorophyll analysis

Chlorophyll content was measured based on the Arnon method (Arnon, 1949). Fresh leaf sample (0.5 g) was ground in liquid nitrogen and added to 10 mL of pre-chilled 80% acetone and mixed well. The mixture was centrifuged at 12000 rpm for 10 min and the supernatant was collected. The supernatant was then diluted using 80% acetone and the absorbance was measured at 645 and 663 nm using acetone as blank in a UV-visible spectrophotometer (OPTIZEN 2120UV; Mecasys, Daejeon, Korea). Ten samples per treatment were used for chlorophyll analysis. Total chlorophyll content was calculated using the formula:


$$Total\;chlorophyll\;=\;20.2\;(A_{645})\;–\;8.02\;(A_{663})\;\times \;V/100\;\times \;W$$

Where V = final volume of the extract; W = FW of the sample.

### Biochemical analysis

For the NSC analysis, hot air oven-dried leaves (70°C) were used. NSC analysis was performed using a previously described protocol with modifications (Li et al., 2018). In total, 0.1 g DW^-1^ of powdered leaf and root samples were placed in 10 mL centrifuge tubes, and 5 mL of 80% ethanol was added. The mixture was incubated at 80°C in a water bath shaker for 30 min, and then centrifuged at 3500 rpm for 10 min. The pellets were extracted two more times using 80% ethanol. Supernatants were retained, combined, and stored at 4°C to determine the total soluble sugar content. The ethanol-insoluble pellet was used for starch extraction. Ethanol was removed via evaporation. Starch in the residue was released in 2 mL distilled water for 15 min in a boiling water bath. The solution was cooled to room temperature (22°C) and 2 mL of 9.2 M perchloric acid was added. Starch was hydrolyzed for 15 min and 4 mL distilled water was added to the solution following centrifugation at 4000 rpm for 10 min. The pellets were extracted again using 2 mL of 4.6 M perchloric acid. Supernatants were retained, combined, and made up to 25 mL to determine starch content. The soluble sugar and starch concentrations were measured spectrophotometrically (OPTIZEN 2120UV, Korea) at 620 nm using the anthrone method, and the starch content was calculated by multiplying the glucose concentrations by a conversion factor of 0.9. Glucose was used as the standard. Ten replicates per treatment were used for NSC analysis.

### Proline adjustment

Proline concentration in leaves and roots was determined using a previously described protocol (Forlani and Funck, 2020). A mixture of 0.3 g fresh leaf samples and root samples (freeze stored) and 5 mL sulfosalicylic acid was homogenized and then centrifuged at 3000 rpm for 20 min. The supernatant was mixed with 2 mL glacial acetic acid and 2 mL acid ninhydrin, and the resulting mixture was boiled at 100°C for 25 min in a water bath. After cooling, 4 mL of toluene was added and allowed to settle. The absorbance of the extracts at 520 nm was evaluated using a UV visible spectrophotometer. Ten samples per each treatment were used for proline estimation.

### Lipid peroxidation activity

Lipid peroxidation was evaluated by estimating malondialdehyde (MDA) content. MDA was measured based on a method established (Zhang et al., 2021). A mixture of 0.5 g fresh plant material and 5 mL of 5% trichloroacetic acid was centrifuged at 12000 rpm for 25 min. The supernatant was mixed with 2 mL of 0.67% thiobarbituric acid solution and heated for 30 min at 100°C in a water bath. Sample absorbance at 450, 532, and 600 nm was measured using a blank containing all reagents. Ten replicates per each treatment were used for lipid peroxidation analysis. MDA content in the sample was calculated using the formula:


$$C\;(\mu mol\;g^{-1})\;=\;6.45(A_{532}\;–\;A_{600})\;–\;0.56A_{450}$$

### DPPH antioxidant assay

The antioxidant activity of the extracts was evaluated using 1,1-diphenyl-2-picrylhydrazyl (DPPH) assays (Dao et al., 2012). Briefly, a 0.1 mM solution of DPPH in 90% methanol was prepared and then 1.5 mL of this solution was mixed with 1.5 mL of each sample (crude extract prepared with naturally dried leaf samples) at concentrations of 100, 50, 25, and 10 µg/mL in 90% ethanol. After 30 min incubation in the dark, the decrease in absorbance of the solution at 517 nm was measured spectrophotometrically. DPPH inhibitory activity was expressed as the percentage inhibition (I %) of DPPH in the aforementioned assay system calculated as:


I = (1 – B/A) × 100

Where A and B are the activities of the DPPH without and with test material. The inhibitory concentration at 50% values were calculated from the mean values of data from three determinations. Butylated hydroxyanisole at various concentrations (1, 2.5, 5, and 10 µM) was used as a positive control. Ten replicates per treatment was used in DPPH assays.

### GC/MS analysis conditions

For the VOC analysis the seedling roots for the treatments (control and drought) were harvested, immediately transferred to liquid nitrogen and stored a -80°C until analysis. VOC analysis was performed using a GC/MS-TSQ8000QQQ2014 instrument (Thermoscientific, USA), solid-phase microextraction (SPME, Supelco, PC-420D, USA), SPME fiber assemblies (Supelco, PDMS/DVB/CAR; Sigma–Aldrich, St. Louis, MO, USA), and headspace bottles with caps (22ml, Santa Clara, CA, USA). Freeze-stored seedling root samples were cut into pieces, and sample preparation and headspace solid phase microextraction (HS-SPME) procedure was performed as previously described (Xiang et al., 2014). The seedling roots (10 mg) were transferred to the headspace bottle, which was placed in a dry heat block adjusted to 60°C and then incubated for 20 min. Three measurements were made using at least three replicates for each sample of seedling roots. Reproducibility was indicated by the relative standard deviation. The operation conditions for GC/MS were as follows: VFWAXms (30 m × 0.25 mm (0.25 lm); temperature of 40°C (held for 5 min) and 250°C (held for 5 min) with an increase rate of 5°C min^-1^; the carrier gas was helium (99.999%), which was allowed to flow for 25 min with an inlet temperature of 230°C. The characteristics of the MS system were as follows: ion source of 70 eV EI at a temperature of 200°C; transfer line temperature of 250°C; mass range of 45–450 amu with a collection time of 3–20 min. Four replicates per treatment was used for VOC analysis.

### Identification and quantification of volatile compounds

Volatile substances from the leaf samples were identified by comparing the components of the mass spectra with the MS database (NIST version 2.0) in addition to comparing the sample spectra to those of authentic reference standard (1,2,3-trichloropropane) when required. A previously described peak normalization method was used to determine the relative content of each VOC (Yang et al., 2015). Quantitative analysis in the percentage of each VOC was calculated using the formula below:


Relative content = M/N * 100%

where, M is the peak area of the individual aromatic compounds; N is the total peak area.

### ABA content

Freeze stored root samples were prepared based on a previously established protocol (Liu et al., 2012). Briefly, five replicates of each frozen root sample (approximately 100 mg for each replicate) were ground to a fine power in liquid nitrogen using a mortar and pestle. Each sample was weighed into a 1.5 mL tube, mixed with 750 μL cold extraction buffer (methanol: water: acetic acid, 80:19:1, v/v/v) supplemented with internal standard (10 ng ^2^H_6_ ABA), vigorously shaken on a shaking bed for 16 h at 4°C in dark, and then centrifuged at 13000 rpm for 15 min at 4°C. The supernatant was carefully transferred to a new 1.5 mL tube and the pellet was remixed with 400 μL extraction buffer, shaken for 4 h at 4°C, and centrifuged. The two supernatants were then combined and filtered using a syringe-facilitated 13 mm diameter nylon filter with a pore size of 0.22 μm (Hyundai Micro, Seoul, Korea). The filtrate was dried via evaporation under the flow of nitrogen gas for approximately 4 h at room temperature and then dissolved in 200 μL methanol. The dissolved mixture was used for LC/MS analysis. LC/MS, SPME and GC/MS analysis were performed at the National Instrumentation Center for Environmental Management (NICEM), Seoul National University, Republic of Korea.

### Statistical analysis

All experiments were conducted twice and the results are reported as mean ± SE. Data were analyzed *via* two-factor analysis of variance (ANOVA) using the R program (v.3.5.1). The treatment mean values were compared *via* Tukey’s (least significance difference) test; statistical significance was set at P ≤ 0.05. Pearson correlation coefficient was carried out among the treatments and a heat map was generated with *corrplot* package using the R program (v.3.5.1).

## Results

### Plant growth, water relation, and chlorophyll content

**Figure 1:**
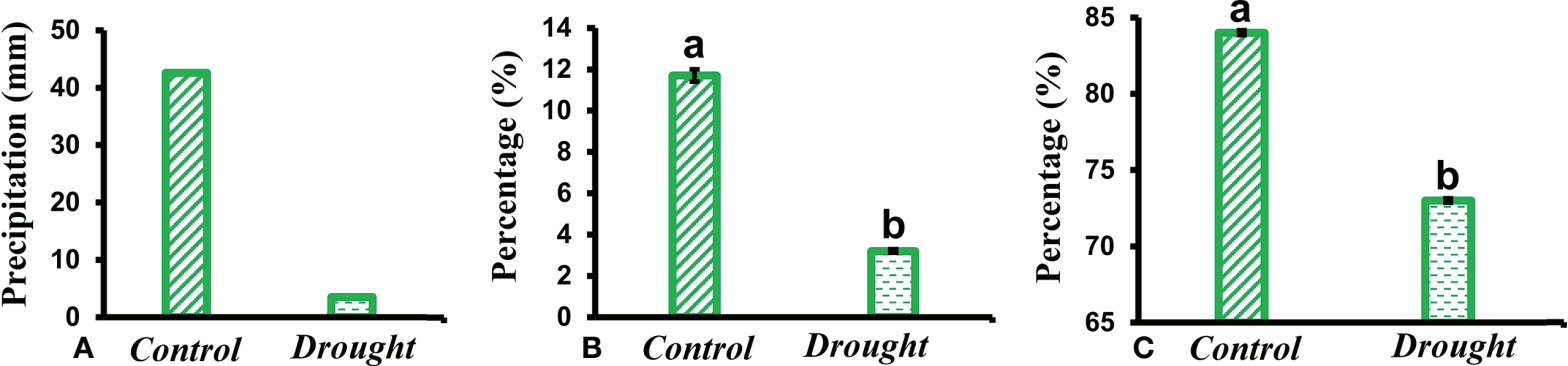
Meteorological data. **(A)** total rainfall received in control and drought plots throughout the study period **(B)** soil moisture content (SMC) measured in soil obtained from control and drought treated pots kept within the Plexiglas roof **(C)** relative water content (RWC) measured in leaves of control and drought stressed plants 32 DAT. Means denoted with different letters indicate significance at p<0.05.

Total precipitation recorded during the study period showed a higher percentage of rainfall received in control plots compared to a minimal amount in treatment plots (**Figure 1A**). The SMC in drought-treated pots (3.2 ± 0.09%) significantly decreased compared to that in control pots (12 ± 0.3%) (**Figure 1B**). RWC is regarded as a measure of the water status in plants, reflecting the metabolic activity in plants. RWC content in leaves of drought-treated plants (72% ± 0.05) significantly decreased compared to that in control plants (84 ± 0.09%) (**Figure 1C**).

**Figure 2:**
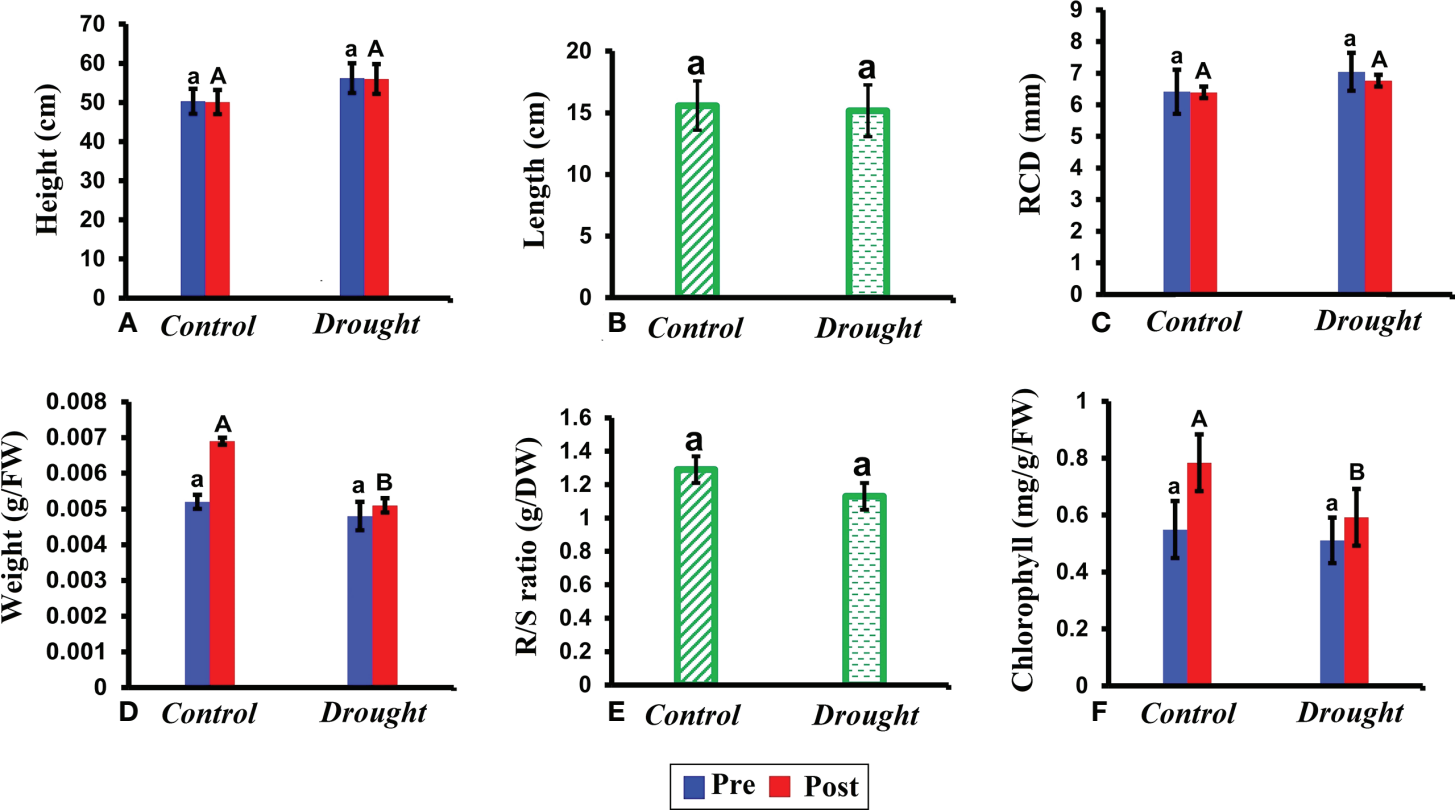
Morphological and physiological responses of *P.strobus* seedlings under drought. **(A)** plant height measured before and after the treatment period 32 DAT **(B)** root length measured in control and drought plants **(C)** root collar diameter (RCD) recorded 32 DAT **(D)** fresh leaf weight recorded before and after 32 DAT **(E)** root/shoot ratio measured after the treatment period **(F)** total chlorophyll content measured in leaves of control and drought plants before and after the treatment period. Results represent that short term severe drought has not caused drastic morphologically changes, rather caused physiological changes in the leaf fresh weight, chlorophyll pigment content and R/S ratio index. Means denoted with different letters indicate significance at p<0.05.

No significant difference was observed in plant height, root length, and root collar diameter between control and drought plants (**Figures 2A–C**). In contrast, drought stress significantly decreased the leaf weight (P< 0.05) (**Figure 2D**). This decrease was evident based on pre and post-treatment values. The leaf weight in control plants increased by 2% after the treatment period whereas that in drought-treated plants increased only by 0.2%. Similar to leaf weight, but a non-significant decline in root/shoot ratio was observed (**Figure 2E**). The chlorophyll content significantly decreased in the leaves of drought-treated plants compared to that in control plants (**Figure 2F**).

### Non-structural carbohydrate changes

**Figure 3:**
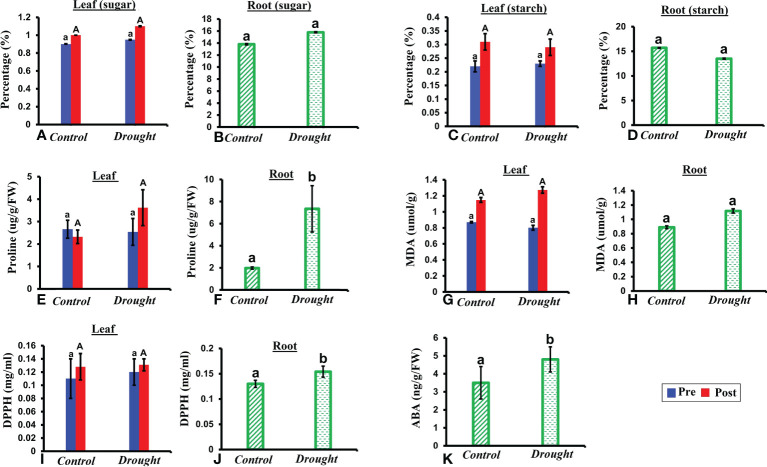
Biochemical responses of *P.strobus* seedlings to drought. **(A, B)** total soluble sugar content in leaves and root of control and stressed plants **(C, D)** total starch content in leaves and roots 32 DAT **(E, F)** proline content in leaves and root samples of control and drought plants **(G, H)** lipid peroxidase activity-MDA content in leaves and root samples of control and drought plants **(I, J)** antioxidant assay measured *via* DPPH content in root and leaf samples 32 DAT **(K)** ABA content in roots 32 DAT. Results denote that severe drought significantly influences root proline and DPPH activity compared to a non-significant increase in leaves. In addition, a non-significant increase is observed in sugar (leaf and root), lipid peroxidase activity (leaf and root) with a non-significant decrease noted for starch content (leaf and root). Means denoted with different letters indicate significance at p<0.05.

NSCs represent major substrates in plant metabolism and have been implicated in mediating drought-induced tree mortality (Signori-Müller et al., 2021). A non-significant increase in soluble sugar content was observed in both leaves and roots in drought-treated plants (**Figures 3A, B**). In contrast, a non-significant decrease in starch content was observed in both leaves and roots in drought-treated plants compared to that in the control plants 32 DAT (**Figures 3C, D**). The decrease in starch content was 1.5% higher in roots than that in leaves.

### Proline content

Free proline functions as an important osmoprotectant during abiotic stress. A non-significant increase in free proline level was observed in leaves of drought-treated plants compared to that in leaves of control plants (**Figure 3E**). A significant accumulation of free proline was observed in roots of drought-treated plants compared to that in control plants 32 DAT (P< 0.05; **Figure 3F**). This accumulation was 5-fold higher than that in the control plants.

### Lipid peroxidation

MDA content is an indicator of membrane lipid peroxidation, which reflects the extent of damage at adverse conditions. A non-significant increase in MDA content was observed in both the leaves and roots of drought-treated plants at 32 days (**Figures 3G, H**). Therefore, the oxidative damage caused to roots was greater (1-fold increase) than that in leaves during drought treatment.

### Antioxidant changes

Since the majority of natural antioxidants possess reactive hydrogen atoms, which serve as reductants, the DPPH assay is a good measure of the standard antioxidant profile. High antioxidant activity was observed in roots compared to that in leaves at 32 DAT (**Figures 3I, J**). A non-significant increase in antioxidant activity was observed in drought-treated leaves compared to that in control leaves (**Figure 3I**). In contrast, a significant increase (2-fold) in antioxidant activity was observed in drought-treated roots compared to that in the control.

### Effect of drought stress on root ABA and VOC composition

**Figure 4:**
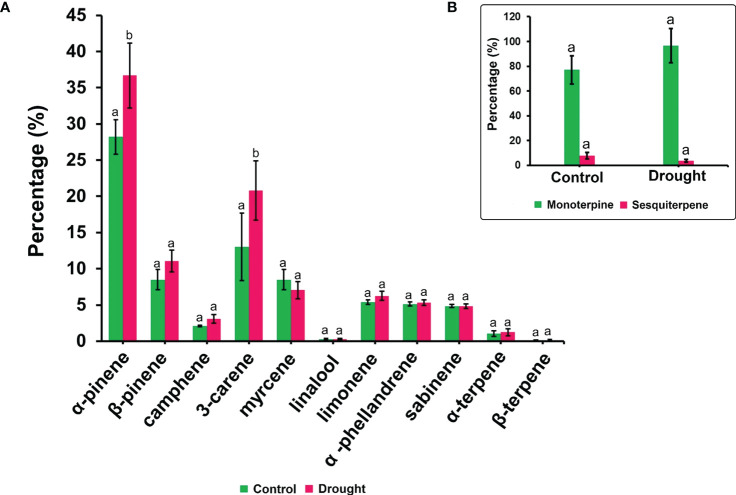
Volatile synthesis (VOC)-Monoterpenes. **(A)** major monoterpenes and their content in root samples **(B)** total accumulation of main monoterpenes and sesquiterpenes in root samples measured 32 DAT. Results indicate the influence of severe drought on the significant accumulation of two critical monoterpenes α-pinene and delta-3-carene apart from a non-significant increase for other critical monoterpenes like β-pinene, camphene and limonene. Significant accumulation of total monoterpene content is noted compared to drought treated seedling samples. Means denoted with different letters indicate significance at p<0.05.

**Figure 5:**
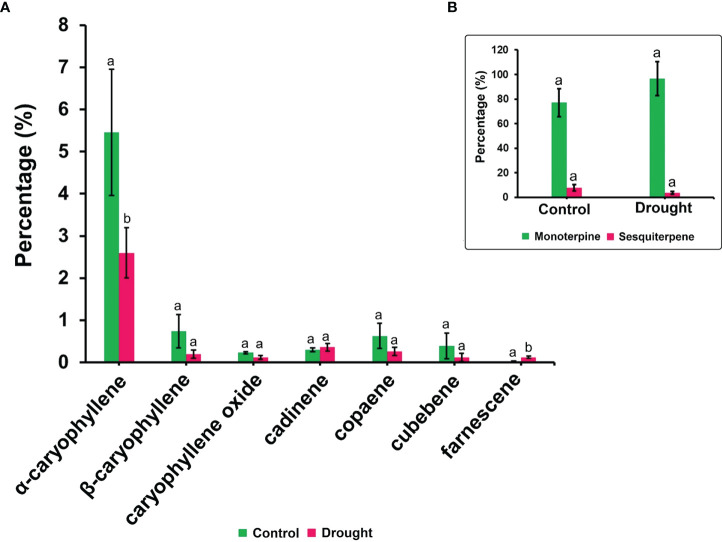
Volatile synthesis (VOC)-Sesquiterpenes. **(A)** list of sesquiterpenes and their content in root samples **(B)** total accumulation of main monoterpenes and sesquiterpenes in root samples measured 32 DAT. Results highlight the influence of severe drought on the decline in total sesquiterpene accumulation supported by a decrease in major sesquiterpenes like α-caryophyllene, β-caryophyllene and cadinene. Farnescene being the only sesquiterpene showing a non-significant increase in their content among drought treated seedling sample. Means denoted with different letters indicate significance at p<0.05.

**Figure 6:**
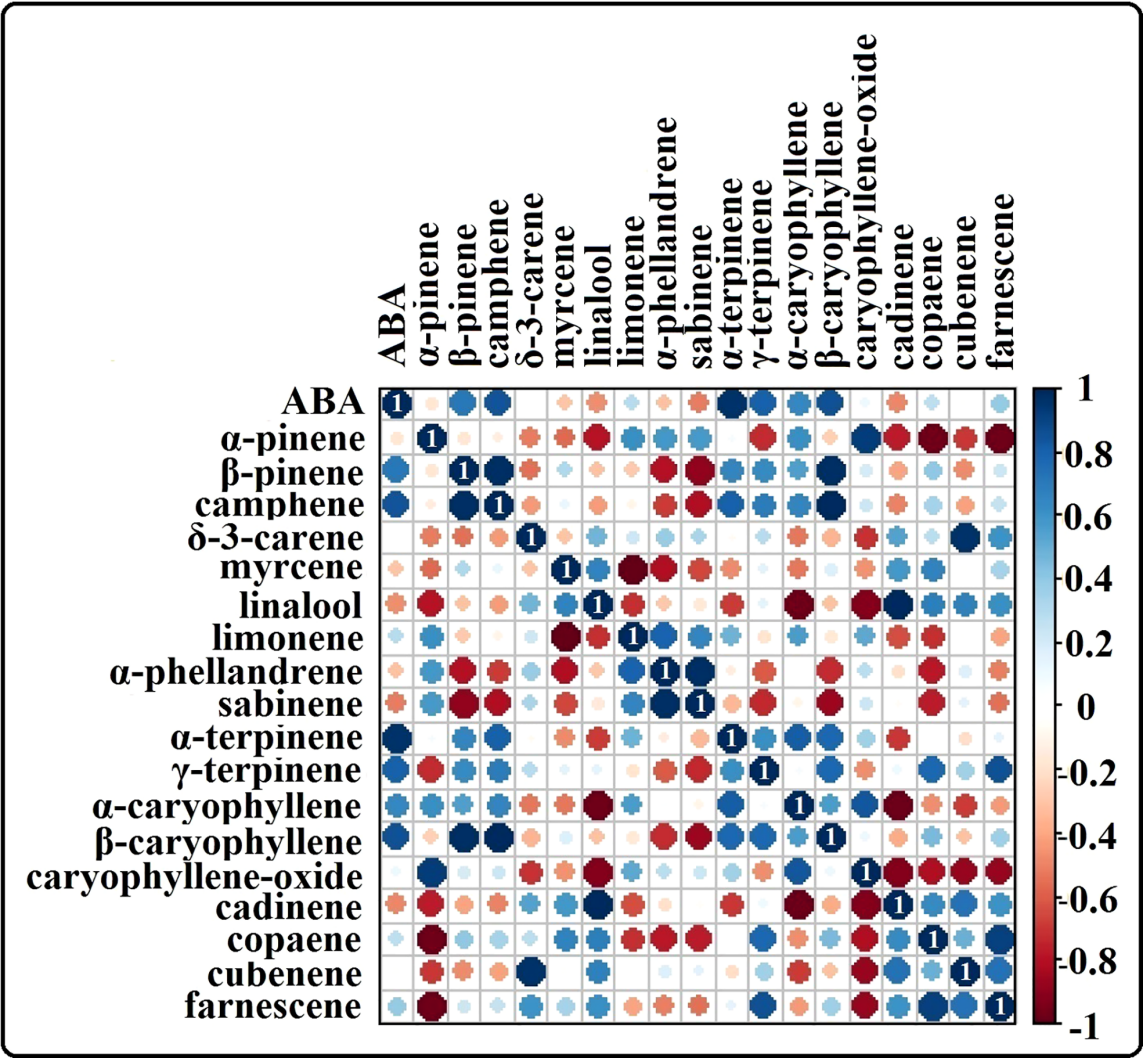
Heat map showing the correlation analysis of ABA content vs terpene content from our study. Positive correlation between ABA and the terpenes are shown in ‘blue’ and negative correlation between ABA and terpenes are shown in the color range ‘red’. Results highlight a strong positive correlation between several monoterpenes like α-pinene, camphene, α-terpene, y-terpinene and ABA whereas negative/weak positive correlation is observed for several sesquiterpenes like caryophyllene oxide, cadinene, copaene, cubenene and farnescene in relation with ABA.

The phytohormone ABA is a key signal in drought response. ABA concentrations in drought-treated plant roots showed a significant increase (1.2-fold) compared to that in control plant roots (**Figure 3K**). Analysis of the spectrum for each VOC and subsequent data verification allowed identification of several types of VOCs in the control and severe drought-treated plants. Few of the commonly occurring VOCs after drought stress included several monoterpenes and sesquiterpenes (**Figures 4**, **5**). In total, 11 types of monoterpenes (including α-pinene, β-pinene, D-limonene, and α-phellandrene) and 7 types of sesquiterpenes (including cadinene, α and β-caryophyllene, copaene, and caryophyllene oxide) were predominantly detected in our study (**Figures 4**, **5**). The relative contents of monoterpenes like α-pinene and 3-carene were significantly higher in drought-treated plants than in control plants (**Figure 4A**). The relative content of other monoterpenes including β-pinene, camphene, linalool, limonene, α-phellandrene, sabinene, α-terpene, and β-terpene showed a non-significant increase, with myrcene being the only monoterpene showing a non-significant decline in content compared to that in control plants (**Figure 4A**). Overall, the total monoterpene content was relatively higher in drought-treated plants (94%) than in control plants (78%) (**Figure 4B**). In contrast, the relative content of total sesquiterpenes reduced to 2% in drought-treated plants compared to 5% in control plants (**Figure 5B**). A major sesquiterpene, α-caryophyllene content showed a significant decline in drought-treated plants (**Figure 5A**). Farnescene was the only sesquiterpene showing a significant increase in content in drought-treated plants compared to that in control plants at 32 DAT (**Figure 5A**). The contents of other sesquiterpenes, including β-caryophyllene, caryophyllene oxide, cadinene, copaene, and cubene, showed a non-significant decrease in drought-treated plants (**Figure 5A**). A strong negative correlation (cadinene), weak positive correlation (copaene, cubenene, farnescene) and a strong positive correlation (α-caryophyllene, β-caryophyllene) was observed between ABA and sesquiterpenes profiles (**Figure 6**). On the other hand, a strong positive correlation (β-pinene, camphene, α-terpinene, *γ*-terpinene) and a weak negative correlation (α-pinene, myrcene, linalool, limonene, α-phellandrene, sabinene), was found between ABA and monoterpene profiles (**Figure 6**). A list of all the other volatiles including alcohols, terpenes, esters, ketones and aldehydes identified in our study have been listed in the **Supplementary Table S1**.

## Discussion

Drought causes morphological and physiological changes in plants. The drought response is primarily determined by the rate and extent to which water status in plants is hydraulically regulated (Skelton et al., 2015). Subsequently, this phenomenon mediates the duration and intensity of the physiological changes and the processes that underlie mortality. In our study, the significant decrease in SMC in drought-treated plants was primarily due to the lack of water supply (only 20% received). SMC decline also indicates a lack of nutrient uptake by *P. strobus* plants under drought condition. Similarly, several forest tree species have been reported to show low SMC and nutrient uptake under severe drought conditions (Chakhchar et al., 2018; Ji et al., 2021). Monitoring the SMC deficits can indicate potential impacts on *P. strobus* development and soil health, supporting the assessment of drought-tolerant, resilient, and vulnerable ecosystems. Leaf RWC is an important indicator of water status in plants as it relates to the balance between leaf water supply and transpiration rate (Tanentzap et al., 2015). Decline in RWC in *P. strobus* plants indicates a lower stomatal conductance and photosynthetic exchange under water deficit conditions. Supporting the RWC evidence, the lower chlorophyll content observed in leaves of drought-treated plants indicates an impact on chlorophyll abundance. Impacts on chlorophyll pigment, especially photosystem II, under drought conditions has been reported in various tree species (Batra et al., 2014; Li et al., 2020; Vastag et al., 2020; Javed et al., 2021). For instance, teak plants under severe drought exposure (20 days of water withholding) show a reduction in chlorophyll content and leaf RWC by 9.57% (Galeano et al., 2019).

Plants regulate their R/S ratios in response to the availability of substrates (mainly water) and to environmental changes. Our findings (non-significant decline in drought-treated plants) suggest that a drought-induced shift in R/S ratio can improve plant water uptake potential in a short window determined by both water and atmospheric parameters. Similar to our results, opposite effects of R/S water balance have been previously reported in two grass species (Gargallo-Garriga et al., 2014). An opposite metabolic activity in shoots and roots may account for the lack of large reductions in productivity during drought experiments at least for a short term (Gargallo-Garriga et al., 2014; Boudiar et al., 2020). An intra-specific variation in assimilate partitioning (water/soluble sugar) between roots and shoots has been found in wheat during drought stress (Fang et al., 2017; Mathew et al., 2019).

Plants produce and accumulate compatible solutes to improve water absorption and maintain hydration of protoplasts under drought stress (Blum, 2017). In the present study, the accumulation of organic solutes, including soluble sugar and proline, indicates their role as osmoregulants to prevent leaf senescence and improve *P. strobus* plant performance under drought condition. The increase in proline level is in parallel with a low RWC in the leaves of drought-treated *P. strobus* plants. Several studies have suggested that proline accumulation in plants can be considered as a general response to abiotic stresses (Yaish, 2015; Ghosh et al., 2022). In contrast, soluble sugars may also act as osmoprotectants based on their substitution with water *via* hydrogen linkage and by establishing bonds with proteins and membranes and they provide protection against dehydration or peroxidation (Ahmad et al., 2020). Our results indicated that an increased proline concentration and accumulation of high concentration of soluble sugars in the leaves and roots protect *P. strobus* plants from intercellular oxidative damage. Proline has been recognized as a signaling molecule that activates ROS detoxification pathways (Anwar Hossain et al., 2014).

Notably, our findings support the hypothesis that drought stress decreases the starch concentration with an increase in soluble sugar concentration (Dai et al., 2018; Tsamir-Rimon et al., 2021). Severe drought treatment largely reduced photosynthetic carbon (C) assimilation as observed in the chlorophyll analysis (**Figure 2**). The restricted C assimilation affected the C supply chain and cannot satisfy the C demand of *P. strobus* plants for maintaining metabolism and growth. This resulted in depletion of internal C reservoirs of starch (**Figure 4**). Starch depletion under water deficit conditions, owing to depleted photosynthesis, has been also found across different species and tissues (Regier et al., 2009; Mitchell et al., 2013; Klein et al., 2014; Lloret et al., 2018). Similar to our results, the trends of starch depletion and increase in sugar levels have been previously observed in another Pinus species, Pinus radiata (Mitchell et al., 2013). Under drought treatment, NSCs buffer the asynchrony between C supply and growth, which decrease and increase the C demand needed to maintain a minimum level of plant respiration (Sperling et al., 2017; Ryhti et al., 2022). More importantly, we speculate that the starch depletion observed in our study may lead to mortality in P. strobus plants over long drought periods. Future studies should elucidate whether the duration of drought alone determines gross depletion of carbohydrates (starch) leading to mortality.

In recent years, several studies have investigated the signaling function of VOCs in the ecosystem (Vivaldo et al., 2017; Ninkovic et al., 2021). Our results showed that drought stress had a positive effect on the production of monoterpenes, as the levels were higher than those in control plants (**Figure 4**). The alternate C source (starch depletion) can contribute to monoterpene production under drought conditions, preventing these isoprenoids from being reduced (de Souza et al., 2018). The other possible reason may be the increase in soil temperature, which in turn favors monoterpene synthesis as the internal monoterpene synthesis respond to increase in temperature (Birami et al., 2021). Increase in leaf monoterpene emissions from *P. halepensis* and *Cistus albidus* has been attributed to monoterpene accumulation within leaves which is favored during drought periods (Turtola et al., 2003). Water stress induces a shift in terpene composition when water deficit exceeds 4 days (Ormeño et al., 2007). The patterns in monoterpene synthesis in P. strobus plants over prolonged water deficit conditions needs to be examined in future studies. Notably, monoterpene accumulation, especially α-pinene, in roots causes oxidative stress leading to growth inhibition (Singh et al., 2006). Therefore, future studies should investigate whether an increase in α-pinene synthesis leads to additional oxidative damage in P. strobus roots, thereby aggravating drought stress.

Reduced levels of sesquiterpenes in drought-treated plants might have been due to the shift in terpene composition as our study period exceeded a month. Our results suggested that sesquiterpenes may probably be replaced by monoterpenes when drought is prolonged, because drought can impede the cyclization of sesquiterpene precursors (Kopaczyk et al., 2020; Arizmendi et al., 2022). The increase in ABA content and the subsequent stomatal closure is also considered an important signaling event under drought stress across several species (Bharath et al., 2021; Rehman et al., 2021). The increase in root ABA content positively correlates with an increase in several monoterpene and negatively correlates with several sequiterpene content in our study. Although certain studies reported the link between ABA and isoprenoid synthesis under drought (Perreca et al., 2020), whether an increase in ABA content triggers monoterpene synthesis under drought stress remains to be studied. Drought stress has been found to decrease the accumulation of ABA via regulation of the methyl salicylate pathway (Jin et al., 2021). Notably, the production of isoprene and terpenoids is stimulated by jasmonic acid (JA) in plants (Tanaka et al., 2014). Interestingly, the JA and salicylic acid (SA) responsive signalling cascades mediated by JASMONATE ZIM DOMAIN PROTEIN (JAZ) and MYC2 transcription factors can enhance the emission of constitutively emitted isoprene, monoterpenes and sesquiterpenes, which may upregulate stress-specific hormonal signaling (Yang et al., 2019; Dani and Loreto, 2022). Therefore, it will be interesting to study the crosstalk among phytohormones associated with the synthesis of root VOCs in P. strobus seedlings, especially the pathway existing between terpene synthesis and ABA response under water-deficit conditions. The analyzed correlation coefficient results provided novel insights on the internetwork among volatiles (terpenes) and plant hormone ABA under severe drought stress. Controlling water transpiration *via* stomata represents one of the main strategies for plants to increase drought tolerance. This movement is strictly regulated by various environmental stimuli, such as water status, light, and CO_2_ concentration, as well as the endogenous factor, ABA (Daszkowska-Golec and Szarejko, 2013; Driesen et al., 2020). The significant increase in root ABA levels in our study denotes the signaling of roots to fasten the stomatal closure, thereby reducing the transpiration water loss.

## Conclusion

Severe drought drastically affected the water transport from roots to leaves (early stomatal closure) and disrupted the chlorophyll content in P. strobus plants. The impaired chlorophyll functioning subsequently limited the C assimilation process because the intake of atmospheric C is reduced. Consequently, the production of energy (glucose) is reduced in P. strobus plants. Under severe drought, the soil temperature increases and prevents the invasion of soil microbes and P. strobus plants produced relatively higher amounts of root monoterpenes. However, the synthesis of isoprenes in plants requires a surplus of C, which is limited owing to water deficit conditions. Therefore, C for producing energy required for VOC production (monoterpenes) in P. strobus plants is obtained from the internal storage reservoir (starch). Since the monoterpene α-pinene can induce oxidative stress, we speculate that P. strobus plants may undergo both drought and oxidative stress. This in turn causes a significant increase in ABA and proline accumulation in roots. However, whether monoterpenes also signal the leaf stomatal closure via ABA is currently unknown. To maintain intact plant cells and reduce the oxidative damage, P. strobus plants produce additional osmolytes in the form of sugar and proline. Together, our results suggest that starch depletion is an indicator for plant mortality in P. strobus under prolonged exposure to severe drought conditions.

## Data availability statement

The raw data supporting the conclusions of this article will be made available by the authors, without undue reservation.

## Author contributions

UC and HK designed the experiment. UC performed the field, laboratory experiments and wrote the manuscript. SB and SK assisted with laboratory experiments. KK, CP, AH Y-SL assisted with field experiments. All authors contributed to the article and approved the submitted version.

## Funding

This work was jointly supported by a grant from National Institute of Ecology (NIE), funded by the Ministry of Environment (MOE), Republic of Korea (NIE-B-2022-02) and basic science research program through National Research Foundation (NRF) funded by the Ministry of Education (2021R111A2044159), Republic of Korea.

## Conflict of interest

The authors declare that the research was conducted in the absence of any commercial or financial relationships that could be construed as a potential conflict of interest.

## Publisher’s note

All claims expressed in this article are solely those of the authors and do not necessarily represent those of their affiliated organizations, or those of the publisher, the editors and the reviewers. Any product that may be evaluated in this article, or claim that may be made by its manufacturer, is not guaranteed or endorsed by the publisher.

## Abbreviations

ABA, abscisic acid; C, carbon; DAT, days after treatment; DPPH, 1,1-diphenyl-2-picrylhydrazyl; MDA, malondialdehyde; NSC, non-structural carbohydrate; ROS, reactive oxygen species; RWC, relative moisture content; SMC, soil moisture content; VOC, volatile organic compound.
