# Supplementary material for: Short-term severe drought influences root volatile biosynthesis in eastern white pine (Pinus strobus L)
Source: Front Plant Sci. 2022 Oct 26;13:1030140. doi: 10.3389/fpls.2022.1030140 (PMC9644029; doi:10.3389/fpls.2022.1030140)
Supplement: Supplementary file 1 [file DataSheet_1.doc]

**Supplementary Material**

**Short-term severe drought influences root volatile biosynthesis in eastern white pine *(Pinus strobus L)***

Umashankar Chandrasekaran^a,d^, Siyeon Byeon^a^, Kunhyo Kim^a^ , Seo Hyun Kim^a^, Chan Oh Park^a^, Ah reum Han^c^*, Young-Sang Lee^c^ and Hyun Seok Kim^a,b,d,e^,*

***correspondence**

[cameroncrazies@snu.ac.kr](mailto:cameroncrazies@snu.ac.kr)

subalphine@nie.re.kr

**
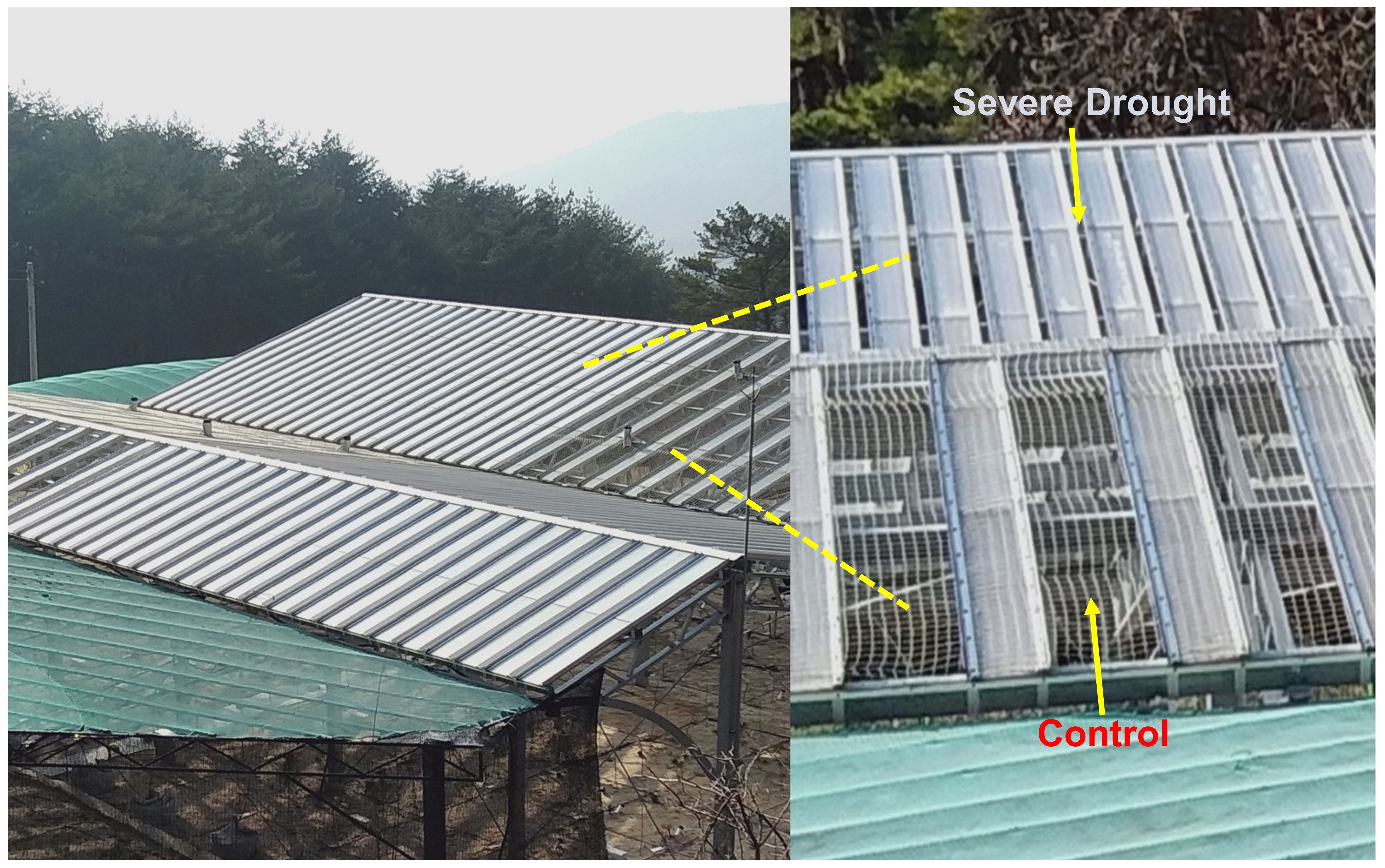
**

**Figure S1** Experimental setup of our field study at the Mt.Jiri, Gurye Jeongnam Province, Republic of Korea. Transparent Plexiglas roof tops (at a height of 3 m) controlled the amount of natural precipitation: 100% and 20% of the roof area was open for control and SD treatments, respectively. The pot seedlings were kept exactly below the opening for the control and severe water deficit study. The experiment was carried out for 32 days.

**
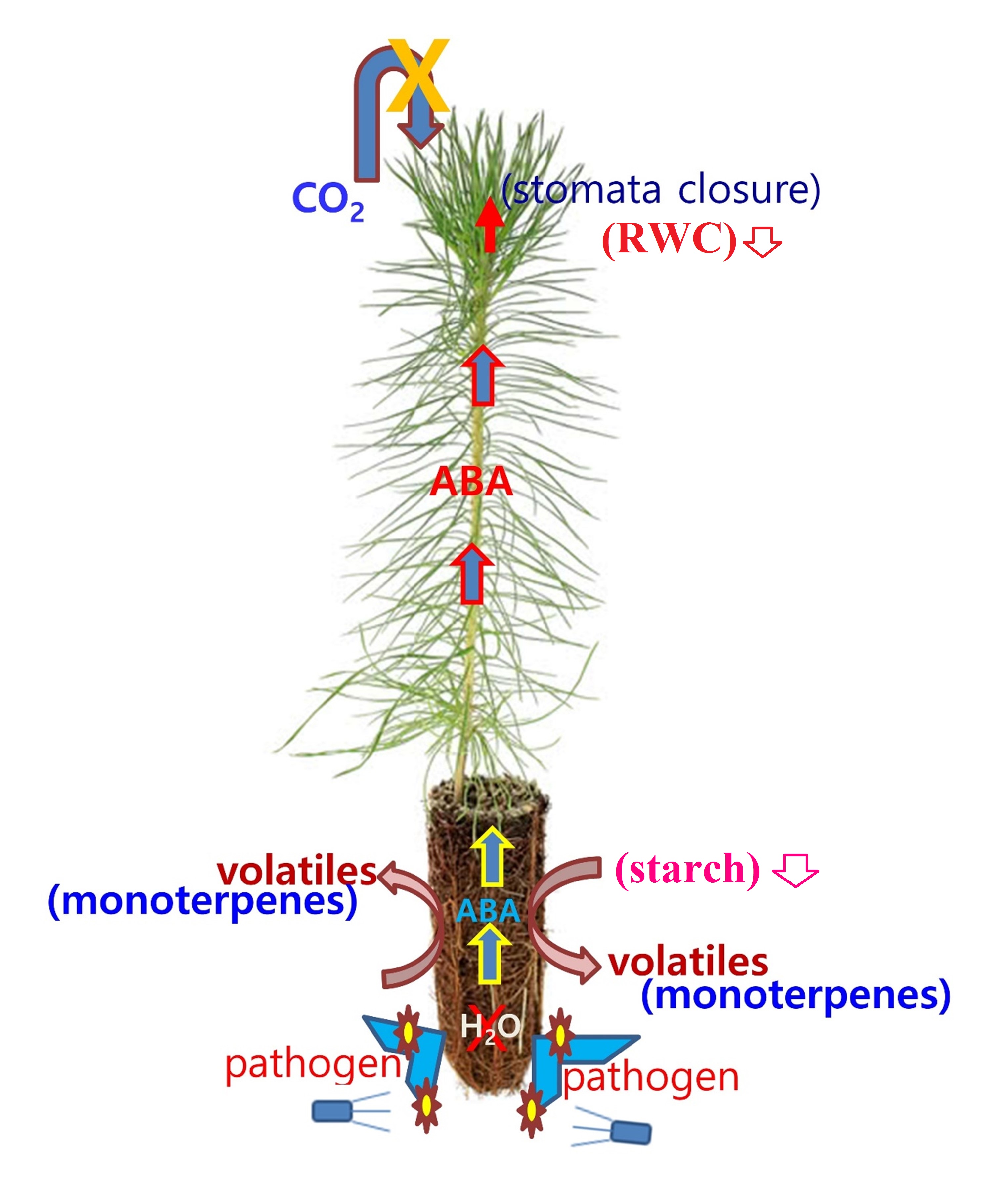
**

**Figure S2 Graphical abstract of the responses of *P.strobus* seedlings to short-term severe drought.** Drought leads to dry soil, which causes roots to signal leaf stomata closure *via* ABA, that in turn limits atmospheric carbon intake. This results in the shortage of internal carbon supply for the synthesis of root monoterpenes. The carbon shortage is balanced by the breakdown of energy (carbon) from internal storage reservoirs ‘starch’. Continuous utilization of starch reservoirs might lead to *P.strobus* seedling mortality.

**Table S1. Identification of root volatile components in *Pinus strobus* seedlings (mean ± SD)**

| **No** | **Components** | **RT** | **RI*** | **Relative content (%)** | **Relative content (%)** |
| --- | --- | --- | --- | --- | --- |
|  |  |  |  | **Control** | **Drought** |
| 1.  2.  3.  4.  5.  6.  7.  8.  9.  10.  11.  12.  13.  14.  15.  16.  17.  18.  19.  20. | ***Alcohols***  (Z)-3-Hexen-1-ol  Linalool  1-octen-3-ol  Terpinen-4-ol  Fenchyl alcohol  4-terpineol  Myrtenol  Methyleugenol  3-ally-6-methoxyphenol  Torreyol  4-methyl-1-(1-methylethyl)-3-cyclohexen-1-ol  4-trimethyl-3-cyclohexane-1-methanol  3,7-dimethyl-1,6-octadien-3-ol  6,6-dimethyl-2-methylene-bicyclo[3.1.1]heptan-3-ol  6,6-dimethyl-bicyclo[3.1.1]hept-2-ene-2-methanol  P-menth-1(7)-en-9-ol  2-methoxy-4-(2-propenyl)-phenol  1,6-dimethyl-4-(1-methylethyl)-1-napthalen-ol  ***Terpenes***  Tricyclene  Isoledene | 25.57  30.96  27.66  33.74  38.34  38.34  43.91  52.74  58.96  59.73  33.74  38.34  30.96  36.45  43.91  45.28  58.96  59.72  11.79  31.9 | 938  938  943  913  930  926  887  938  884  911  922  925  929  913  909  825  876  884  919  914 | 0.38 ± 0.06  0.29 ± 0.09  0.5 ± 0.02  0.16 ± 0.01  0.74 ± 0.06  0.74 ± 0.06  0.29 ± 0.03  0.32 ± 0.1  0.25 ± 0.1  0.15 ± 0.01  0.16 ± 0.01  0.74 ± 0.06  0.29 ± 0.09  0.19 ± 0.01  0.29 ± 0.03  0.1 ± 0.03  0.25 ± 0.1  0.15 ± 0.01  0.24 ± 0.02  0.12 ± 0.04 | ND  0.5 ± 0.02  0.2 ± 0.02  0.15 ± 0.02  0.76 ± 0.1  0.76 ± 0.1  0.17 ± 0.05  0.17 ± 0.03  ND  0.19 ± 0.03  0.15 ± 0.02  0.75 ± 0.1  0.5 ± 0.02  0.17 ± 0.02  ND  ND  ND  0.19 ± 0.03  0.4 ± 0.08  0.2 ± 0.03 |

**Table S1. Identification of root volatile components in *Pinus strobus* seedlings (mean ± SD) (cont..)**

| **No** | **Components** | **RT** | **RI*** | **Relative content (%)** | **Relative content (%)** |
| --- | --- | --- | --- | --- | --- |
|  |  |  |  | **Control** | **Drought** |
| 21.  22.  23.  24.  25.  26.  27.  28.  29.  30.  31.  22.  33.  34.  35.  36.  37.  38.  39.  40.  41.  42.  43.  44. | ***Terpines***  Gurjunene  Murrolene  α-terpinene  Terpinolene  Humulene  2,6,6-trimethylbicyclo[3.1.1]hept-2-ene  o-cymenene  tridecane  tetradecane  Atis-16-ene  1-methyl-4-(1-methylethylidene)-cyclohexene  Naphthalene  Undecane  1-methyl-4-(1-methylethyl)-1,3-cyclohexadiene  1-methyl-3-(1-methylethyl)-1,3-benzene  o-isopropenyltoulene  2,3-dimethoxytoulene  1,7,7-trimethyl-bicyclo[2.2.1]hept-2-ene  1-methyl-2-(1-methylethyl)-1,3-benzene  1-tert-butyl-2,5-dimethoxy-benzene  1,2-dimethoxy-4-(2-propenyl)-benzene  Methyl(1-methylethyl)-benzene  7,11-dimethyl-3-methylene-1,6,10-dodecatriene  3,7,11-trimethyl-1,3,6,10-dodecatraene | 31.9  40.36  18.33  18.33  18.33  12.32  21.84  22.09  22.09  63.74  18.33  40.36  14.64  18.33  21.84  27.83  36.26  19.07  21.84  47.28  52.74  21.84  36.26  38.64 | 895  926  926  917  945  941  940  946  945  820  920  934  948  914  939  929  851  900  941  973  914  936  864  879 | 0.15 ± 0.05  0.39 ± 0.06  0.25 ± 0.01  0.83 ± 0.4  0.37 ± 0.06  23.52 ± 2.0  0.48 ± 0.02  0.78 ± 0.09  0.78 ± 0.09  0.22 ± 0.02  0.37 ± 0.06  0.37 ± 0.08  1.24 ± 0.11  0.37 ± 0.06  0.48 ± 0.02  0.55 ± 0.03  0.25 ± 0.05  4.25 ± 0.01  0.48 ± 0.02  0.52 ± 0.05  0.32 ± 0.1  0.48 ± 0.02  ND  ND | 0.2 ± 0.03  ND  0.27 ± 0.02  0.27 ± 0.02  0.27 ± 0.02  24.38 ± 7.7  0.58 ± 0.08  0.31 ± 0.09  0.31 ± 0.09  0.19 ± 0.01  ND  0.32 ± 0.1  0.76 ± 0.1  0.27 ± 0.02  0.58 ± 0.08  0.66 ± 0.07  ND  0.4 ± 0.03  0.58 ± 0.08  0.42 ± 0.1  0.17 ± 0.03  0.41 ± 0.05  0.11 ± 0.03  0.87 ± 0.3 |

**Table S1. Identification of root volatile components in *Pinus strobus* seedlings (mean ± SD) (cont..)**

| **No** | **Components** | **RT** | **RI*** | **Relative content (%)** | **Relative content (%)** |
| --- | --- | --- | --- | --- | --- |
|  |  |  |  | **Control** | **Drought** |
| 45.  46.  47.  48.  49.  50.  51.  52.  53.  54.  54.  55.  56.  57.  58.  59.  60.  61.  62.  63.  64.  65. | ***Terpines***  Para-mentha-1,4(8)-diene  α-pinene  β-pinene  camphene  delta-3-carene  myrcene  limonene  α-phellandrene  sabinene  α-terpinene  y-terpinene  α-caryophyllene  β-caryophyllene  caryophyllene oxide  cadinene  copaene  cubenene  farnescene  ***Esters***  Bornyl acetate  Fenchyl acetate  Myrtenyl acetate  6,6-dimenthylbicyclo[3.1.1]methyethyl carbonate | 37.12  15.61  17.56  13.97  12.32  17.56  19.07  17.77  19.48  18.33  20.83  33.94  37.52  52.18  42.23  28.36  28.36  36.26  32.98  32.98  38.15  38.15 | 817  817  923  944  932  936  928  927  936  930  916  946  944  899  926  898  918  850  934  929  833  832 | ND  28.2 ± 2.4  8.5 ± 1.4  2.1 ± 0.1  13.03 ± 4.67  8.5 ± 1.4  5.37 ± 0.3  5.13 ± 0.3  4.85 ± 0.2  1.05 ± 0.4  0.11 ± 0.06  5.46 ± 1.5  0.74 ± 0.4  0.23 ± 0.03  0.3 ± 0.05  0.63 ± 0.3  0.39 ± 0.3  0.02 ± 0.01   - 1. ± 0.15   0.79 ± 0.2  0.17 ± 0.04  0.15 ± 0.05 | 0.2 ± 0.1  36.7 ± 4.5  11.04 ± 0.02  3.1 ± 0.6  20.82 ± 4.11  7.04 ± 1.2  6.26 ± 0.6  5.3 ± 0.4  4.84 ± 0.3  1.23 ± 0.5  0.16 ± 0.06  2.6 ± 0.6  0.2 ± 0.1  0.12 ± 0.04  0.36 ± 0.09  0.26 ± 0.1  0.12 ± 0.1  0.125 ± 0.03  1.16 ± 0.4  ND  0.16 ± 0.02  0.4 ± 0.1 |

**Table S1. Identification of root volatile components in *Pinus strobus* seedlings (mean ± SD) (cont..)**

| **No** | **Components** | **RT** | **RI*** | **Relative content (%)** | **Relative content (%)** |
| --- | --- | --- | --- | --- | --- |
|  |  |  |  | **Control** | **Drought** |
| 66.  67.  68. | ***Ketones***  2,2,3,6-tetramethyl-2,3 dihydro-4-furo[2,3-b]pyran-1  ***Aldehydes***  Benzaldehyde  dodecanal | 47.28  31.12  39.02 | 978  928  961 | 0.52 ± 0.05  0.11 ± 0.02  0.37 ± 0.07 | ND  ND  0.11 ± 0.03 |

*ND- not detected;*

*RT-retention time;*

*RI*-Retention indices found in literature and database (NIST version 2.0)*

**Table S2. Results of two-factor ANOVA (control x drought) in leaf, root, sugar, starch, R/S ration, proline, MDA, DPPH, RWC and SMC content in *Pinus strobus*** seedlings subjected to severe drought for 32 days.

| **Variable** | **Treatment** | **df** | **p-value** |
| --- | --- | --- | --- |
| 1. ABA 2. Sugar(root) 3. Sugar(leaf) 4. Starch(root) 5. Starch(leaf) 6. Proline(leaf) 7. Proline(root) 8. Fresh weight(leaf) 9. Plant Height 10. MDA(leaf) 11. MDA(root) 12. Chlorophyll 13. SMC 14. RCD 15. R/S ratio 16. DPPH(leaf) 17. DPPH(root) 18. RWC | Control x drought  Control x drought  Control x drought  Control x drought  Control x drought  Control x drought  Control x drought  Control x drought  Control x drought  Control x drought  Control x drought  Control x drought  Control x drought  Control x drought  Control x drought  Control x drought  Control x drought  Control x drought | 1  1  1  1  1  1  1  1  1  1  1  1  1  1  1  1  1  1 | 0.043*  0.261  0.0247*  0.286  0.102  0.767  0.049*  0.0093**  0.467  0.459  0.766  0.039*  0.012*  0.981  ***  0.999  0.0103*  *** |

*Significance levels are indicated as p<0.001 (***); p<0.01 (**) p<0.05(*). ABA-Abscisic acid; MDA-Monoaldehyde; SMC-Soil moisture content; RCD-Root collar diameter; R/S ratio-root/shoot ratio; DPPH-2,2-Diphenyl-1-picrylhydrazyl; RWC-Relative water content*
